# Early Life
*From an address delivered before the Bristol Medico-Chirurgical Society on 10th November, 1948.


**Published:** 1949-04

**Authors:** A. V. Neale

**Affiliations:** Professor of Child Health in the University of Bristol


The Bristol
Medico-Chirurgical Journal

*A Journal of the Medical Sciences for the*

West of England and South Wales

*" Scire est nescire, nisi id me
Scire alius sciret."*

APRIL, 1949

### EARLY LIFE*

BY

A. V. Neale, M.D., F.R.C.P.

*Professor of Child Health in the University of Bristol*

At the passing of the first Public Health Act in 1848 the infant mortality in *the industrial areas of some of our big cities* was about 400 per thousand live births, perhaps a maximum for all time in this country. To-day we see the great skill and care devoted to the health and safety of mother and child, with the result that infant mortality is now one-tenth of that figure, and there has been a like decrease in the maternal risks. Good team-work yields increasingly good results, and in some areas—notably in Bristol—the saving of infant life is highly efficient. With this background of reasonable but not complacent satisfaction, we can approach the future with confidence, but must remember that our population problems are complex and that significant effects must follow the recent decline in the birthrate. With these matters our economic future is bound up.

Antenatal pædiatrics is a somewhat new approach to our problem. In 1900 Ballantyne made a start on antenatal care which can be considered the beginning of the organized services now available. But in 1926, a year before his death, he warned us against over-zealous action and emphasized the need for the most careful discretion :

* From an address delivered before the Bristol Medico-Chirurgical Society on 10th November, 1948.

reminding us that the aim at all times must be natural labour and the welfare of the baby. He laid emphasis on the need for continuity in the care to be maintained throughout the whole service for mother and child. This is very pertinent in view of the arrangements under the new Health Act: we shall await with interest the effects of the new plans on the health of mother and baby.

### ANTENATAL INFLUENCES

The fœtus *in utero* is subject to its own special environment. We must recognize three phases: (A) The early formative phase, up to the twelfth week; (B) a middle phase of simple body-expansion; and (C) the later weeks of storage and maturation.

Susceptibilities differ at each stage. Influences are greatest in the first stage of rapid growth. At its most rapid rate of growth, the weight of the embryo increases 28,000 times in a week: its length at 4 weeks is 4 mm., at 8 weeks 28 mm., and at 12 weeks 75 mm. (3 in.). The ovum, which weighs 0.005 mg., develops into the differentiated organism weighing about 3,000 grams, this resulting from 44 successive cell-divisions and having its greatest velocity in the first 8 weeks of prenatal life. (Warkany, 1947.) The nutritional and oxygen requirements for this high rate of growth are normally ensured by rapid and liberal placental development. We can contrast this with the deterioration of placental efficiency in the later weeks of pregnancy.

The hæmochorial type of placentation in the human is of special interest because it makes for unusually easy transference from maternal to fœtal circulation. This may facilitate passive immunity by antibodies: but it may also produce increased vulnerability and reactions to Rh and other maternal-fœtal incompatibilities. The fœtus, especially in the period 4–12 weeks, is a very susceptible structure, at the mercy of infective elements, unfavourable genetic influences, nutritional deficiency, and possibly even placental injury or separation. Virus disease, e.g. Rubella, may have serious effects on the rapidly-differentiating tissues, and may cause congenital defects: deafness, cataract, microcephaly and heart disease. (Swan.)

The operation of genes is as yet outside any control. But it seems that "gene-action" in the fœtus may be conditioned by other factors. There is much in favour of chemical (enzyme) bases for gene action (Gruneberg), possibly modified by maternal nutrition. Gilman has succeeded in producing in rats "genetic" abnormalities by injecting the mother with cresyl blue early in pregnancy: perhaps the first directly successful experiment in distorting fœtal development. Fœtal susceptibilities may be determined, not only by hereditary influences but by the total circumstances of uterine development. Which is better, a good egg in a bad environment or a bad egg in a good environment?

Warkany (1944) was able to demonstrate in rats the considerable frequency of congenital abnormalities in litters born of mothers deficient in vitamins of the $B_2$ group, especially nicotinic acid and riboflavine. The vitamins are probably essential for normal embryonic cell division and the action of tissue-organizers. The most dramatic effects are produced by even a single dose of X-rays. In small animals with short gestation periods, the fœtal tissues are extremely sensitive to radiation : death or grotesque deformity may result (Job). In man, examples of defect—mainly in the nervous system—have arisen from this cause (Engelking).

The wonderful researches of the late Sir Joseph Barcroft have thrown a flood of light on fœtal physiology. Fœtal movements can be initiated as early as the twelfth week. An accurate index of fœtal welfare, they appear early and increase in later months, only to diminish towards full term. The oxygen supply during the last weeks of pregnancy tends to diminish owing to placental changes and the relatively great mass of the fœtus. But the best oxygenated blood from the umbilical vein is directed to the fœtal brain, up to the time of the first breath. The risks of intra-uterine anoxia are thereby partly covered, and the high oxygen capacity of fœtal hæmoglobin is a further help. Fœtal hypoglycæmia is probably rare, but when associated with fœtal anoxia it may cause irreparable damage to the brain. Intra-uterine fœtal hypoxia has not yet received sufficient study as a possible cause of later cerebral palsy or mental disorder.

In the last three months the fœtus acquires two-thirds of its calcium, three-quarters of its protein, four-fifths of its iron, and a store of vitamins (except vitamin A). Nature has apparently decided that vitamin A shall be obtained from the colostrum, which is heavily loaded with carotene—hence its yellow colour.

We do not know exactly what processes initiate normal labour. We do not know in many cases why birth is premature : nor why some babies with a birth weight of 5½ lb. or less go ahead excellently and others encounter great difficulties. Which is the better overall risk : a premature baby of 4 lb. or a post-mature of 12 lb. ? Hereby hangs a pretty problem of relative safety.

Prematurity, immaturity and still-birth are to some extent inter-related. Baird showed in Aberdeen that the incidence is correlated inversely with social status. During special periods of the war prematurity and still-births increased in Leningrad and in Holland : before the war, the rates in depressed areas in Britain compared very unfavourably with the low rates in Holland and New Zealand, where nutrition was exceptionally good. Ebbs (in Toronto) was able to show that improved antenatal care and nutrition had a direct bearing on prematurity as well as on neo-natal health. Asher has suggested that in some cases inferiority of male germ-plasm may be a factor in fœtal inferiority.

Low birth weight is no bar to ultimate physical and mental success. Hess (of Chicago) followed up cases for 25 years : out of 200 infants below 3 lb. at birth, 170 achieved satisfactory development. With particular care for the obstetric risks and the dangers of anoxia a much greater survival-rate among premature babies can be expected. The Bristol quadruplets have shown the valuable results of a combined obstetric-pædiatric team. Miller (at Newcastle) has shown the way to safer care for prematures in the home : an organization including the home service of a skilled and conscientious " neo-natal " sister can produce results nearly as good as may be expected in hospital. Such arrangements should form part of the facilities in each area.

In the antenatal phase the obstetrician is in a special position to assess all the factors which give maximum ease and safety. Eardley Holland made known the real significance of intracranial hæmorrhage in the newborn. The modern use of clinical and radiological data has undoubtedly lessened traumatic effects on the fœtus.

TABLE I

Incidence of traumatic meningeal hæmorrhage and asphyxial intra-cerebral hæmorrhage. (Smith.)

| | Deaths | Per cent. showing : | |
|---|---|---|---|
| | | Tentorial Hæmorrhage | Asphyxial focal Intracerebral Hæmorrhage |
| PREMATURE LIVE-BORN : | | | |
| 1931–35 .. .. | 63 | 22.0 | 9.5 |
| 1941–45 .. .. | 89 | 4.5 | 12.5 |
| FULL-TERM LIVE-BORN : | | | |
| 1931–35 .. .. | 60 | 30.0 | 10.0 |
| 1941–45 .. .. | 103 | 11.5 | 18.0 |

The increase in the asphyxial cases coincides with the wider use of premedication, anæsthetics, etc., which have, on the whole, allowed birth to proceed more slowly. There is ground for discussion in this, but the fact emerges that unskilled use of pain-killing drugs during labour may have serious effects on the baby.

At 36 weeks and onwards there is a diminishing placental efficiency for oxygen, together with increasing fœtal sensitivity to lowered oxygen tension. Periods of fœtal agitation and slowing of the heart-rate may make clear the embarrassment. Maternal anæmia, antepartum haemorrhage, toxæmia, prolonged labour or cord-displacement are some of the additional troubles the baby may have to negotiate. We do not know if oxygen inhalation by the mother is of any service, but correction of anæmia is an urgent necessity.

Antepartum hæmorrhage due to placenta prævia may be responsible for a 30 per cent. overall fœtal morality. But with wise obstetric care the live births can be considerably increased (Mills). The hazards of the fœtus in Cæsarean section can be reduced by swift but gentle action. The natural delivery through the vagina is usually devoid of these hazards. Cerebral and pulmonary œdema may occur. Barcroft was convinced that cutaneous and circumoral contacts stimulated the first breath.

The greatest dangers may exist in the first half-hour of life. The brain-cells may rapidly deteriorate in conditions of oxygen deficiency. Anoxia demands emergency measures. The cause may be single: e.g., cerebral irritation or œdema; or a complex situation may arise from inadequate central response with atelectasis and inhalation of fluids—vernix is particularly harmful; and narcotics may intensify the effect. In anoxia neonatorum there is rapid disturbance of capillary permeability, with œdema of lungs, etc., causing peripheral circulatory failure: anoxia renders the medulla less responsive. The pressing needs are to clear the bronchi by catheter suction, and to give oxygen. Combined skin and circumoral stimulation with, perhaps, careful rhythmical rocking may suffice (Eve). Alpha-lobeline, coramine, etc., have value. There is a critical period of about ten minutes, after which danger is extreme.

In some premature babies apnœic phases recur for some hours: a few minutes' lapse may prove suddenly fatal. The handling of this condition calls for understanding and a skilled programme of action, with the central object of oxygenating the brain.

Table II

Cause of death found in two separate series of a thousand consecutive infant deaths (Potter) per cent.:

| | Series "A" | Series "B" |
|---|---|---|
| Anoxia .. .. .. | 20.0 | 28.5 |
| Primary Prematurity .. | 18.5 | 14.5 |
| Congenital Malformation .. | 14.0 | 11.0 |
| Birth Trauma .. .. | 11.5 | 13.0 |
| | 64.0 | 67.0 |

The Lecturer went on to expound the importance of environment in early life. Mental and physical activities emerge in an orderly plan, and it is possible to assess at any age whether these are normal.

Poor health or nutrition may produce obvious retardation. Breast-feeding is obviously of great importance both for baby and mother: it should not be lightly abandoned from indolence or for mere temporary convenience. An adequate protein intake is essential: milk provides the obvious source. In the very earliest months and years mental development should receive attention: a "difficult" baby may be a frustrated baby. The influences of happy family life are preponderant: problems of later delinquency may be avoided by early attention and care.

### REFERENCES.

Asher, C.—*Arch. Dis. Child.*, 1948; **23**, 142.

Baird, D.—*Jour. Obst. and Gynæc.*, 1945; **52**, 217. *Ibid.: Lancet*, 1948; **2**, 531.

Ballantyne, J. W.—*Brit. Med. Jour.*, 1901; **1**, 813. *Ibid: Trans. Edin. Obstet. Soc.*, 1906; **3**, 33.

Barcroft, J.—*Prenatal Life*, Oxford, 1946.

Ebbs, J. H.—*Jour. Nutrit.*, 1941; **22**, 515.

Engelking, E.—*Klin. Monatsbl. f. Augenr*, 1935; **94**, 151.

Eve, G. C.—*Brit. Med. Jour.*, 1948; **2**, 554.

Gruneberg, H.—*Animal Genetics and Medicine*, London, 1947.

Job, T. T., *et al.*—*Am. Jour. Anat.*, 1935; **56**, 97.

Miller, F. J. W.—*Arch. Dis. Child.*, 1947; **22**, 54.

Mills, W. G.—*Brit. Med. Jour.*, 1948; **2**, 896.

Potter, E. L.—*Jour. Am. Med. Assoc.*, 1944; **124**, 336.

Smith, C.—*Advances in Pœdiatrics*, London, 1947; **2**, 46.

Swan, C.—*Med. Jour. Australia*, 1945; **1**, 122.

Warkany, J.—*Jour. Nutrit.*, 1944; **27**, 477. *Ibid.: Advances in Pœdiatrics*, London, 1947; **2**, 1.

